# A Physics-Informed Manifold Neural Operator Framework for Multi-Parameter Prediction of Polymer Aging in HTPB Solid Propellants

**DOI:** 10.3390/polym18111400

**Published:** 2026-06-04

**Authors:** Shun Liu, Hongfu Qiang, Tingjing Geng, Xueren Wang, Shudi Pei, Xin Ju

**Affiliations:** National Key Laboratory of Solid Rocket Propulsion, PLA Rocket Force University of Engineering, Xi’an 710025, China; liushunhanzo@163.com (S.L.);

**Keywords:** solid propellant, accelerated thermal aging, crosslink density, physics-informed learning, neural operator, manifold learning

## Abstract

Predictive modeling of thermal aging in solid propellants is challenging because HTPB-based propellants are highly filled particle-reinforced polymer systems with sparse experimental data, nonlinear parameter coupling, and partially unclear aging mechanisms. This study proposes a Physics-Informed Manifold Neural Operator (PIMANO) framework for multi-parameter prediction of polymer aging in HTPB solid propellants. Accelerated thermal aging, stress relaxation, and swelling experiments were used to obtain aging temperature, aging time, crosslinking density, and viscoelastic Prony-series parameters. A continuous aging-state field was first reconstructed over the temperature–time domain by radial basis function interpolation. Crosslinking density was then introduced as a physically interpretable bridge-state variable linking aging conditions with viscoelastic responses. Among three candidate kinetic models, the modified Arrhenius–Avrami model gave the best fitting performance for crosslinking-density evolution, with R^2^ = 0.988 and MRE = 0.0199. By combining local multi-scale neighborhood features, manifold latent representations, and DeepONet-based operator learning, PIMANO established a unified mapping from aging conditions to multi-parameter viscoelastic responses while incorporating bridge-state consistency, parameter non-negativity, and evolution-direction constraints. Under the RBF-augmented validation setting, PIMANO-ae achieved RMSE = 0.7847, MAE = 0.3366, R^2^ = 0.9995, and MRE = 0.0027. Compared with the traditional model, RMSE, MAE, and MRE were reduced by 94.93%, 96.47%, and 96.85%, respectively. Temperature leave-one-out validation further yielded average R^2^ values of 0.9469–0.9647 and MRE values of 4.98–6.21% at unseen aging temperatures. These results demonstrate that PIMANO provides an accurate, stable, and physically interpretable framework for multi-parameter aging prediction and life-assessment modeling of polymer-based energetic materials.

## 1. Introduction

HTPB-based solid propellants are highly filled particle-reinforced polymer systems in which the polymer binder forms the continuous phase, while oxidizer and metal fuel particles constitute the dominant dispersed reinforcement. Although the polymer phase accounts for only a limited fraction of the total composition, it plays a central role in particle bonding, interfacial stress transfer, viscoelastic relaxation, and aging-sensitive constitutive evolution of the composite propellant [1]. During long-term storage and service, polymer aging in such systems involves a series of coupled processes, including thermo-oxidative aging, post-curing, chain scission, interfacial damage, plasticizer migration, and residual stress redistribution [2]. These processes continuously modify the crosslinked network, alter the relaxation spectrum, and ultimately affect the macroscopic mechanical performance of the propellant. For HTPB-based composite propellants, crosslink density is not only a key microscopic indicator of network evolution, but also directly affects modulus, maximum elongation, and relaxation time scales. Therefore, establishing a unified mapping among aging conditions, polymer state, and constitutive parameters is a central issue in propellant life assessment and digital characterization.

In recent years, the aging behavior of AP/HTPB and related propellant systems has been systematically studied from multiple perspectives, including accelerated thermal aging, thermo-mechanical coupled aging, degradation of dynamic mechanical properties, aging under constant-strain service conditions, and characterization of crosslinking reactions by low-field NMR [3,4,5]. These studies indicate that propellant aging is not a simple change in a single performance index. Instead, it results from the coupled evolution of crosslink density, free volume, relaxation behavior, and macroscopic mechanical response [6,7,8]. Therefore, relying only on a single empirical life criterion or one mechanical index is often insufficient to reveal the actual aging pathway of propellants.

Traditionally, the lifetime prediction of solid propellants mainly relies on high-temperature accelerated tests and Arrhenius extrapolation. This framework has a clear thermo-activation basis and remains one of the most widely used engineering approaches for lifetime evaluation [9,10]. Meanwhile, crosslink density is usually estimated from swelling tests using the Flory–Rehner theory [11]. Time-temperature equivalence is commonly described by the Williams-Landel-Ferry (WLF) equation [12]. Stress relaxation curves are often identified using the generalized Maxwell model [13]. Together, these approaches provide an important mechanistic foundation from three complementary aspects, namely aging kinetics, polymer characterization, and viscoelastic constitutive identification.

However, when the goal shifts to the unified prediction of multiple output parameters under small-sample conditions, the limitations of traditional methods become evident. Arrhenius-type models are more suitable for describing the thermo-activated evolution of a single indicator, but they cannot directly establish a high-dimensional coupled mapping between multiple constitutive parameters and aging states [9,11]. The WLF equation and generalized Maxwell model can identify constitutive parameters, but they cannot automatically learn the nonlinear relation between crosslink state and constitutive parameters [12,13]. In addition, when the number of samples is limited and the experimental points are irregularly distributed, parameter-by-parameter fitting often leads to physically unacceptable outputs.

In recent years, physics-informed neural networks (PINNs), neural operators, and hybrid mechanism–data modeling methods have opened new directions for modeling complex material systems. By embedding physical constraints into the loss function, PINNs improve physical consistency under small-sample conditions [14,15,16,17]. Neural operator frameworks, such as DeepONet, can learn mappings between function spaces and thus provide greater flexibility for high-dimensional and path-dependent problems [18,19,20]. At the same time, research in materials informatics and physics-guided machine learning is moving beyond pure black-box prediction toward paradigms that emphasize mechanistic embedding, interpretability, and structural priors [17,21,22,23]. These developments are particularly relevant to polymer aging in solid propellants, where experimental samples are scarce, mechanisms are intertwined, and multiple constitutive parameters evolve in a coupled manner.

Nevertheless, existing methods still have clear limitations. Pure PINNs are better suited for forward and inverse problems constrained by known governing equations, but they may not be efficient for multi-output mapping of material parameters from irregular experimental samples [14,15,16,17]. Pure neural operators have strong generalization ability, but without a bridge state consistent with material aging mechanisms, they may easily degenerate into high-dimensional black-box mappings [18,19,20]. Fully empirical data-driven models, on the other hand, struggle to capture the physical links among crosslink density, post-curing, and relaxation spectrum evolution [7,8]. Therefore, a more reasonable strategy is not to choose between a mechanistic model and a data-driven model, but to develop a hybrid framework centered on a physical bridge state, while also integrating local data geometry and neural-operator representation capability.

Here we present a physics-informed manifold neural operator framework, PIMANO, for the multiparameter prediction of solid propellant aging. Instead of directly learning a black-box mapping from aging conditions to material responses, PIMANO introduces a physically interpretable bridge state to connect aging history with the evolution of viscoelastic properties. By coupling kinetic-guided state construction with local-state-field representation, manifold-aware latent organization and neural operator learning, the framework is designed to capture both the intrinsic aging trajectory and the strong coupling among material parameters in the small-data regime. This strategy moves beyond the conventional separation between mechanism-based modeling and data-driven prediction, and provides a unified route for embedding physical structure into operator learning. More broadly, the present work establishes a general paradigm for aging-state characterization in complex polymer-based energetic materials, with implications for reliability assessment and long-term service prediction.

## 2. Experiments and Aging Kinetics Analysis

### 2.1. Experimental Overview

**Figure 1:**
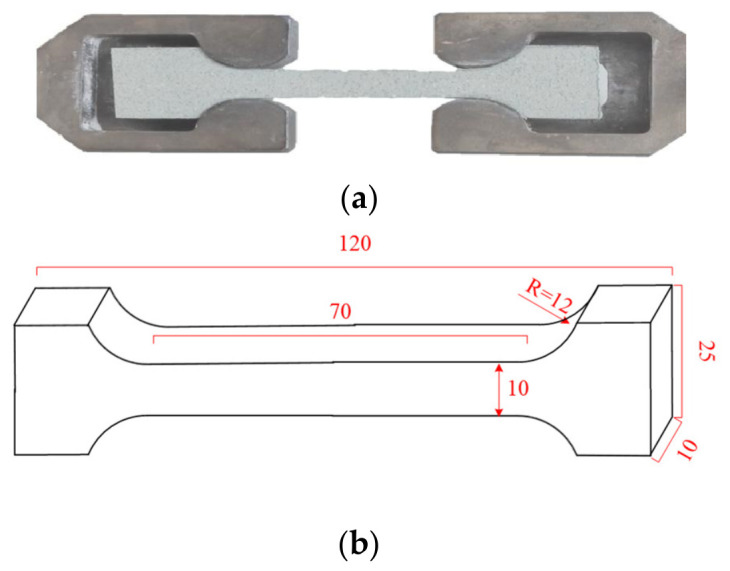
Dumbbell-shaped HTPB propellant specimen: (**a**) photograph of the HTPB propellant specimen; (**b**) schematic of specimen dimensions.

The data used in this study were obtained from accelerated thermal aging tests, stress relaxation tests, and swelling measurements of solid propellants. HTPB propellant specimens were first prepared as square slabs and then reshaped into standard dumbbell-shaped samples according to the Type-B uniaxial tensile specimen dimensions specified in GJB 770B-2005 [24] Test Methods for Propellants, as shown in Figure 1.

Accelerated thermal aging tests were conducted in accordance with QJ 2328A-2005 [25] Test Method for High-Temperature Accelerated Ageing of Composite Solid Propellants. The aging temperatures were set at 50, 60 and 70 °C. The sampling times under different aging conditions are listed in Table 1.

After aging, the specimens were subjected to stress relaxation tests at different temperatures to obtain stress decay curves under different aging conditions. These relaxation curves were then used for parameter identification to determine the Prony-series parameters and the constants in the Williams–Landel–Ferry (WLF) equation. The crosslink density of the HTPB propellant was measured according to the QJ 1616-89 [26] Test Method for Crosslink Density of Composite Solid Propellants.

### 2.2. Determination of Material Parameters

At each aging state, stress relaxation tests were performed on the propellant specimens to obtain the stress decay curves under different aging conditions. To characterize the stress relaxation behavior of the propellant, a fourth-order generalized Maxwell model was adopted in this study. This model consists of one equilibrium spring connected in parallel with four Maxwell branches. The relaxation modulus can be expressed as Equation (1):

(1) $$E\left(t\right)=E_{\infty }+\sum_{i=1}^{n}E_{i}e^{-\frac{\Delta t}{\tau_{i}}}$$

where $E_{\infty }$ is the equilibrium modulus, $E_{i}$ is the modulus associated with the *i*-th Maxwell branch, and $\tau_{i}$ is the corresponding relaxation time. Under a constant strain $\varepsilon_{0}$, the stress relaxation response can be written as Equation (2):

(2) $$\sigma \left(t\right)=\varepsilon_{0}\left[E_{\infty }+\sum_{i=1}^{n}E_{i}e^{-\frac{\Delta t}{\tau_{i}}}\right]$$

Meanwhile, the mechanical response of HTPB propellant exhibits significant time–temperature equivalence. In this paper, the Williams–Landel–Ferry (WLF) equation is adopted in the traditional constitutive characterization to describe the time-scale shift caused by temperature changes, and its basic form is shown in Equation (3).

(3) $$\log_{10}\alpha_{T}=-\frac{C_{1}\left(T-T_{s}\right)}{C_{2}+\left(T-T_{s}\right)}$$

where $\alpha_{T}$ is the temperature shift factor, T is the current temperature, $T_{s}$ is the reference temperature, and $C_{1}$ and $C_{2}$ are material constants. By fitting the stress relaxation curves under different aging states, the constitutive parameters of the propellant as functions of temperature and aging time were obtained. The final parameters for all samples are listed in Table 2. The stress relaxation modulus master curves were obtained according to GJB 770B-2005, Method 413.4, ‘Stress relaxation modulus master curve—uniaxial tension method.’ The fourth-order generalized Maxwell model was then used to parameterize the obtained relaxation spectrum in the form of a Prony series. It should be noted that Prony-series fitting may be ill-conditioned and the resulting Maxwell parameters are not mathematically unique. Therefore, the identified parameters are regarded as effective constitutive parameters associated with the selected model order and testing protocol, rather than unique material constants.

The Prony-series parameters and WLF constants were identified by least-squares fitting of the averaged experimental relaxation responses. This procedure was used to obtain representative effective viscoelastic parameters for subsequent PIMANO modeling, rather than to perform statistical inference of constitutive parameters. Confidence intervals were not estimated because reliable uncertainty quantification would require an additional framework involving measurement-error assumptions, residual-distribution analysis, parameter covariance estimation, and weighting of repeated measurements. Moreover, Prony-series fitting may be ill-conditioned, with different combinations of modulus coefficients and relaxation times producing similar relaxation curves. Therefore, confidence intervals estimated without a dedicated uncertainty-identification procedure could be misleading. The fitted parameters are thus regarded as effective parameters associated with the averaged experimental response and selected model formulations.

### 2.3. Crosslinking Density Aging Kinetics Model

During the thermal aging of solid propellants, the binder matrix, crosslinked network, oxidation reactions, filler/binder interface, and possible plasticizer migration collectively affect the crosslinking density of the material. In general, the crosslinking density does not necessarily change monotonically with aging time. In the early stage of aging, continued reactions of residual functional groups, oxidative crosslinking, or post-curing processes further densify the network structure, resulting in an increase in crosslinking density. In the middle and late stages of aging, backbone scission, crosslink bond fracture, interfacial debonding, or migration of low-molecular-weight components weaken the effective network structure, leading to a decrease in the apparent crosslinking density. Consequently, the crosslinking density of solid propellants often exhibits a non-monotonic evolution characterized by an initial increase, followed by a plateau or a decrease.

For processes dominated by thermal aging, the evolution of material state variables is often described using an Arrhenius thermal aging relationship. Let the characteristic aging rate constant be $K\left(T\right)$; then Equation (4) is obtained.

(4) $$K\left(T\right)=A\exp\left(-\frac{E_{a}}{RT}\right)$$

Among them, *A* is the pre-exponential factor, *E_a_* is the apparent activation energy, *R* is the gas constant, and *T* is the thermodynamic temperature. This temperature dependence originates from Arrhenius’s classic work on the relationship between reaction rate and temperature [10]. Based on this, three candidate evolution models are established in this paper for bridging state variables such as crosslinking density, and the original experimental data are fitted and compared.

The Arrhenius–Avrami model can be regarded as coupling the Arrhenius temperature-dependent rate constant with the Avrami kinetic kernel function, with its theoretical foundation rooted in Avrami’s series of classic papers on phase transformation kinetics [27,28,29]. It is shown in Equation (5):

(5) $$\upsilon \left(T,t\right)=\upsilon_{0}+\left(\upsilon_{\infty }-\upsilon_{0}\right)\left[1-\exp\left({\left(-K\left(T\right)t\right)}^{n}\right)\right]$$

where *n* is the Avrami exponent, used to characterize more complex multistage nonlinear evolution processes.

The traditional Avrami model can essentially only describe a ‘growth–saturation’ process and cannot capture the decrease in crosslinking density caused by chain scission, degradation, or network destruction after long-term thermal aging of solid propellants. Therefore, a temperature-dependent degradation term is introduced, resulting in the modified Arrhenius–Avrami model shown in Equation (6):

(6) $$\upsilon \left(T,t\right)=\upsilon_{0}+\left(\upsilon_{\infty }-\upsilon_{0}\right)[1-\exp({\left(-K\left(T\right)t\right)}^{n})]-K_{d}(T)t$$

The model consists of two physical contributions superimposed: the first term is an Avrami-type crosslinking growth term, and the second term is an Arrhenius-controlled linear degradation term.

**Figure 2:**
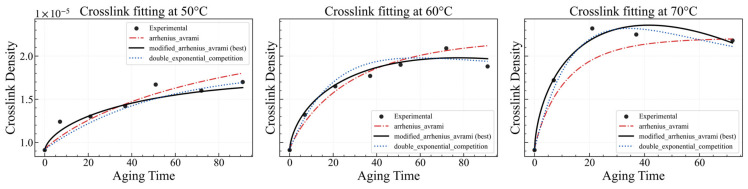
Results of crosslinking density fitted by traditional methods at 50 °C, 60 °C, and 70 °C.

The degradation term in the modified Arrhenius–Avrami model adopts a linear form, which is suitable for describing damage effects that accumulate approximately linearly within the experimental time window. However, from a more general perspective of aging kinetics, both crosslinking growth and degradation damage may themselves be processes that gradually approach saturation. For instance, the number of reactive functional groups is limited, so crosslinking growth cannot increase indefinitely; meanwhile, the network structure that can undergo scission or oxidation also possesses a finite damage capacity. Therefore, a double-exponential competition model can be further adopted, in which both crosslinking growth and degradation weakening are expressed as exponential kinetic processes with saturation characteristics. The double-exponential competition model is written as Equation (7):

(7) $$\upsilon \left(T,t\right)=\upsilon_{0}+A_{1}\left[1-\exp\left({\left(-K_{1}\left(T\right)t\right)}^{n}\right)\right]-A_{2}\left[1-\exp\left({\left(-K_{2}\left(T\right)t\right)}^{n}\right)\right]$$

where *A*_1_ represents the maximum contribution amplitude of the crosslinking growth process to the crosslinking density, and *A*_2_ represents the maximum reduction amplitude of the degradation process to the crosslinking density. By comparing the MRE, and R^2^ metrics of the three candidate models on the original experimental data points (see Table 3), the optimal aging kinetics expression can be selected. The fitting results for crosslinking density are shown in Figure 2.

**Figure 3:**
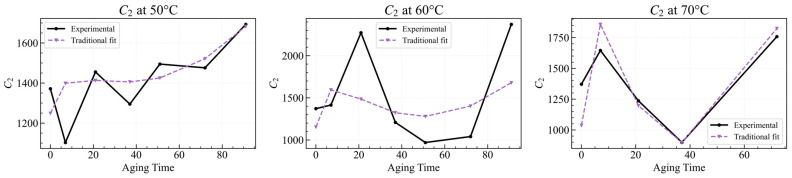

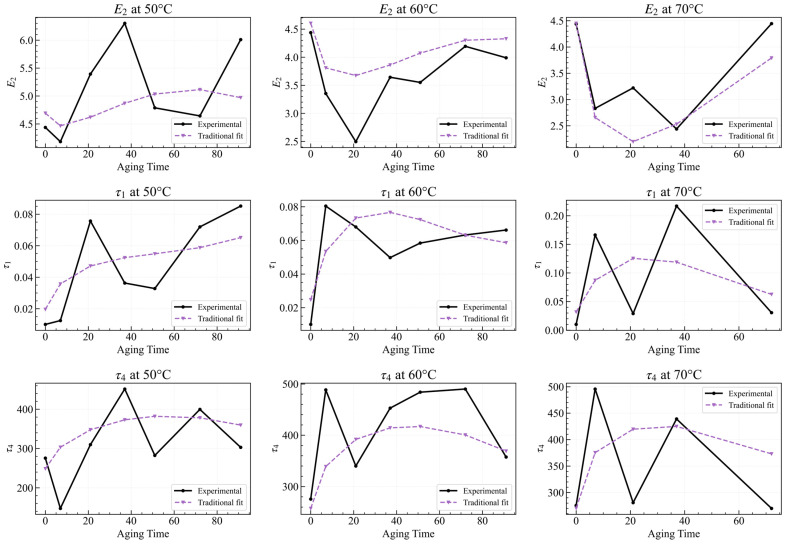
Comparison between traditional fitting results of typical material parameters and experimental values.

Although the Arrhenius, WLF, and generalized Maxwell models provide solid mechanistic interpretation frameworks from the perspectives of aging kinetics, time–temperature equivalence, and viscoelastic constitutive behavior, respectively, these traditional methods still exhibit significant limitations when the research objective shifts toward unified prediction of multiple output parameters under small-sample conditions. The Arrhenius-type equation is more suitable for describing the thermally activated evolution of a single characterization quantity and struggles to directly represent the coupled mapping among multiple sets of constitutive parameters—as shown in Figure 3—making it difficult to capture complex nonlinear trends. While the WLF and Maxwell models can identify curve parameters, they are unable to automatically learn the high-dimensional nonlinear relationship between a crosslinking state and constitutive parameters. Traditional methods generally fail to effectively leverage the local neighborhood geometric structure among experimental data points. Therefore, a unified framework that retains the physical meaning of traditional models while leveraging the high-dimensional mapping capabilities of modern machine learning is needed.

## 3. PIMANO Algorithm Architecture

### 3.1. Overall Architecture

**Figure 4:**
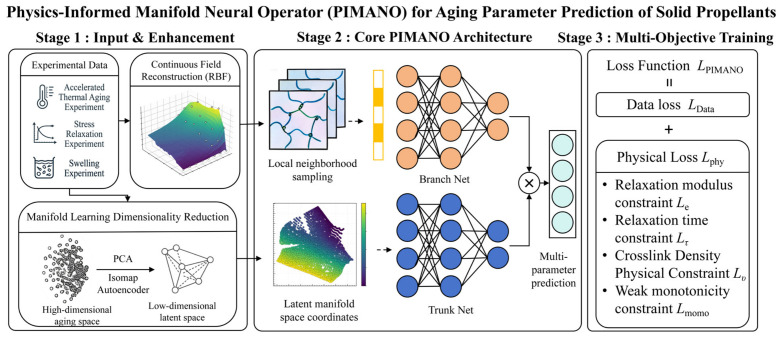
PIMANO algorithm architecture.

The core of multi-parameter prediction for thermal aging of solid propellants is not an ordinary finite-dimensional regression, but rather a continuous nonlinear mapping from the aging-state field to the viscoelastic response parameter field. When the input is limited solely to temperature *T*, aging time *t*, and a single-point crosslinking density *υ*, the model can only learn empirical functional relationships from discrete samples, making it difficult to preserve the coupling structure among parameters, the continuity of the aging trajectory, and the material admissibility constraints. To address this, this paper reformulates the problem as a physics-constrained state-dependent operator learning task: the input consists of the local crosslinking state field, the global aging-manifold coordinates, and the physical bridging states, while the output is a multi-parameter viscoelastic spectral response that satisfies material laws. The complete algorithm flow chart is shown in Figure 4.

The construction and training of the PIMANO model can be summarized into the following four steps:

**Step 1:** Based on experimental measured samples and Radial Basis Function (RBF) augmented samples, a continuous crosslinking density field is constructed. Through neighborhood sampling and feature extraction, a local crosslinking state feature set is obtained, forming the input vector for the branch network.

**Step 2:** Manifold learning dimensionality reduction is applied to the aging-state space to eliminate redundant features and extract the principal path of aging evolution, yielding the manifold coordinates that characterize the global aging process. These coordinates form the extended input matrix for the trunk network.

**Step 3:** Using the operator network (DeepONet) as the foundational architecture, a unified nonlinear mapping model is established from “local crosslinking state field + global aging-manifold coordinates + physical bridging states” to the multi-parameter viscoelastic response spectrum.

**Step 4:** Physical constraints related to thermal aging of solid propellants are embedded, and a composite loss function combining data loss and physics loss is constructed to complete the training and convergence of the model.

Therefore, the overall mapping of PIMANO can be written as Equation (8).

(8) $$y=F\Theta (\upsilon_{\mathrm{loc}}(T,t),z(T,t,c))$$

where Θ represents all trainable parameters, and F denotes the local crosslinking state field. This expression indicates that the main method in this paper is no longer a simple pointwise mapping network, but rather a neural operator that simultaneously couples the local field, low-dimensional manifold, and physical bridging states.

### 3.2. Theoretical Foundation of DeepONet

The theoretical foundation of DeepONet is based on the universal approximation theorem for nonlinear operators, as summarized by Lu et al. [18]. This theorem states that if σ is a continuous non-polynomial activation function, X is a Banach space, $K_{1}\subset X$ and $K_{2}\subset X$ are compact sets, and $V\subset C(K_{1})$ is a compact set, then Equation (9) holds.

(9) $$G:V\to C(K_{2})$$

Let *G* be a continuous nonlinear operator. Then for any $\hat{\varepsilon }$, there exist positive integers n,p,m, constants $c_{i}^{k},\xi_{ij}^{k},\theta_{i}^{k},\zeta_{k}\in \mathbb{R}$, a vector $w_{k}\in {\mathbb{R}}^{d}$, and sensing points $x_{j}\in K_{1}$, such that for any u∈V and any $y\in K_{2}$, the following holds, as shown in Equation (10).

(10) $$\left|G(u)(y)-\sum_{k=1}^{p}\left(\sum_{i=1}^{n}c_{i}^{k}\sigma \left(\sum_{j=1}^{m}\xi_{ij}^{k}u\left(x_{j}\right)+\theta_{i}^{k}\right)\right)\sigma (\omega_{k}\cdot y+\zeta_{k})\right|<\hat{\varepsilon }$$

In the generalized operator approximation theorem further provided by Lu et al. [18], the most commonly used vectorized expression of modern DeepONet is shown in Equation (11):

(11) $$\left|G(u)(y)-\langle \underset{\mathrm{branch}}{\underset{︸}{g(u(x_{1}),u(x_{2}),\ldots ,u(x_{m}))}},\underset{\mathrm{trunk}}{\underset{︸}{f(y)}}\rangle \right|<\hat{\varepsilon }$$

DeepONet is a network in which the branch network outputs all basis coefficients at once, while the trunk network outputs basis vectors of the same dimension, and the final output is obtained via a dot product. This trunk–branch divide-and-conquer structure is an important inductive bias that enables small generalization error, because the two inputs u and y of $G\left(u\right)\left(y\right)$ are physically independent.

What DeepONet provides is not merely a “deeper network”, but a natural formal tool for mapping a local aging-state field to multi-output material responses. However, the original DeepONet only distinguishes between the input function and the output location; it neither incorporates physical bridging states nor explicitly describes the low-dimensional structure of the aging path. Therefore, manifold encoding and physical information are introduced on its basis to form PIMANO.

### 3.3. Manifold Learning Reduced-Order Theory

Although the thermal aging of propellants manifests as high-dimensional coupled variations in temperature, time, crosslinking density, and multiple viscoelastic parameters, its dominant evolution path typically does not fill the entire high-dimensional space but is more likely to lie on a low-dimensional intrinsic manifold. Explicitly extracting this manifold helps improve sample organization, compress redundant degrees of freedom, and enhance the ability of the trunk branch to represent the global evolution position. Three types of methods—PCA, Isomap, and Autoencoder—are employed, and the dimensionality reduction quality is further evaluated using trustworthiness, distance correlation, and reconstruction error.

The basic idea of principal component analysis (PCA) is to find, among all linear projection directions, orthogonal bases that maximize variance, so that the projected low-dimensional coordinates retain as much information from the original data as possible. For the centered sample matrix X, PCA can be formulated as solving the eigenvalue decomposition problem of the covariance matrix, as shown in Equation (12):

(12) $$S=\frac{1}{n-1}X^{T}X$$

Then, the first $d_{z}$ eigenvectors corresponding to the largest eigenvalues are taken to form the projection matrix W. Thus, the two-dimensional latent variables can be written as Equation (13).

(13) $$z_{\mathrm{PCA}}=W^{T}(X-\bar{X})$$

The advantage of PCA lies in its concise form, numerical stability, and strong interpretability, making it particularly suitable for examining whether the material aging trajectory approximately extends along a linear principal direction. If the effects of crosslinking density and aging time are approximately additive, PCA often yields a clear principal evolution axis. However, the limitations of PCA are equally evident: it can only preserve linear correlation structures and struggles to handle nonlinear aging paths characterized by bending, folding, or stage transitions.

Nonlinear Manifold Expansion: Isometric Mapping (Isomap) [30]. When the material state evolution is distributed along a curved manifold, the Euclidean distance between samples no longer reflects their true intrinsic proximity. The core idea of Isomap is to approximate the sample manifold using a neighborhood graph. It calculates the shortest path distance between any two points on this graph, uses it to replace the original Euclidean distance, and then constructs a low-dimensional embedding through classical multidimensional scaling (MDS).

Let the sample set be ${\left\{x_{i}\right\}}_{i=1}^{n}$. First, a *k*-nearest neighbor graph *G* is constructed. Then, the graph geodesic distance matrix $D_{G}=\left[d_{G}\left(i,j\right)\right]$ is obtained. Based on this, the double-centered matrix is as shown in Equation (14):

(14) $$B=-\frac{1}{2}HD_{G}^{2}H$$

where **H** is the centering matrix. Eigendecomposition is then performed to obtain the low-dimensional coordinates. Compared with PCA, Isomap places greater emphasis on preserving the global geometric structure of samples on the manifold, making it particularly suitable for describing nonlinear situations where the same temperature increment corresponds to different state displacements at different aging stages. For propellant aging—a process that may involve the combined effects of crosslinking growth, oxidative chain scission, post-curing, and structural rearrangement—Isomap can more naturally unfold curved trajectories, thereby enhancing the model’s ability to distinguish similarities across different aging stages and identify stage transition points.

Autoencoder (AE) [31] learns a low-dimensional compressed representation of the input data through an encoder $f_{enc}$ and a decoder $f_{dec}$ in the form of a neural network. Its basic form is shown in Equation (15).

(15) $$\begin{array}{cc} z_{\mathrm{ae}}=f_{\mathrm{enc}}(x) & \hat{x}=f_{\mathrm{dec}}(z_{\mathrm{ae}}) \end{array}$$

where $z_{\mathrm{ae}}$ is the AE latent vector and $\hat{x}$ is the reconstructed input vector. By minimizing the reconstruction error, it can be expressed as Equation (16):

(16) $$L_{\mathrm{rec}}={\left\|x-\hat{x}\right\|}_{2}^{2}$$

Unlike PCA, which uses a fixed linear projection, AE can learn highly nonlinear latent representations. This makes it more suitable for handling complex yet continuous material evolution trajectories under small-sample conditions. It is especially advantageous when state variables interact and couple with each other, when local nonlinearities are enhanced, or when different temperature regions correspond to different aging sensitivities. In such cases, the parametric mapping of AE generally offers greater expressive power than traditional manifold methods.

Although the multisource characterization data for thermal aging of solid propellants are high-dimensional, multi-output, and strongly coupled, their main evolutionary patterns may be governed by a few intrinsic variables. Therefore, it is necessary to explicitly extract the low-dimensional geometric structure of the aging path before neural operator learning, and to incorporate this geometric structure as an integral part of PIMANO rather than treating it as an isolated preprocessing step.

### 3.4. Physical Constraints and Composite Loss Design

To ensure prediction accuracy, physical admissibility, and reasonable evolution of internal states, a joint loss function composed of a data fitting term, parameter constraint term, bridging state consistency term, and evolution direction constraint term is constructed for PIMANO. The core idea is to require not only that the model output approximates the experimental labels, but also that the predicted results satisfy the basic physical bounds of viscoelastic parameters and that the predicted bridging states remain consistent with thermal aging kinetics. In this way, physical priors are further advanced from external regularization to the internal representation layers of the network. Since $c_{1}$ is constant with a value of 50, $c_{1}$ is not predicted.

Then the total loss of PIMANO is defined as Equation (17):

(17) $$L_{\mathrm{PIMANO}}=L_{\mathrm{data}}+\lambda_{\tau }L_{\tau }+\lambda_{e}L_{e}+\lambda_{\upsilon }L_{\upsilon }+\lambda_{\mathrm{mono}}L_{\mathrm{mono}}$$

The loss function uniformly adopts the Smooth L1 loss, as shown in Equation (18):

(18) $$L_{\mathrm{data}}={\mathrm{Smooth}\;L1}_{\beta =0.2}(\hat{y},y)$$

where y and $\hat{y}$ are the experimental and predicted multi-parameter response vectors, respectively, and β is the transition parameter of the Smooth L1 loss. Compared to the traditional MSE, this loss function maintains quadratic penalization for small errors and transitions to linear penalization for large errors, making it more robust to local outliers and scale differences that may arise under small-sample conditions. Meanwhile, the variable $\tau_{1}∼\tau_{4}$ is first log-transformed and then standardized to alleviate the training imbalance caused by differences in units and magnitudes among the outputs.

To ensure that the predicted parameters satisfy the basic physical boundaries in the constitutive sense, non-negative constraints are set for the modulus parameters and the relaxation time parameters respectively. For $E_{1}∼E_{4}$, the loss is defined in Equation (19).

(19) $$L_{e}=\frac{1}{N_{e}}\sum_{j\in \{E_{1},\ldots ,E_{4}\}}{\left[\max(0,-{\hat{E}}_{j})\right]}^{2}$$

For $\tau_{1}∼\tau_{4}$, the loss is defined in Equation (20).

(20) $$L_{\tau }=\frac{1}{N_{\tau }}\sum_{k\in \{\tau_{1},\ldots ,\tau_{4}\}}{\left[\max(0,-{\hat{\tau }}_{k})\right]}^{2}$$

Both of the above are forms of soft constraints. Their advantage lies in not disrupting the continuity of backpropagation while encouraging the model to actively stay away from non-physical solution regions during training.

The most critical physical constraint in PIMANO is reflected in the bridging state consistency term. Since the network learns the crosslinking density state $\upsilon_{\mathrm{state}}$ based on $z\left(T,t,\upsilon_{\mathrm{phy}}\right)$, without constraints this crosslinking density state may degenerate into a free latent variable lacking physical meaning. The loss is defined in Equation (21):

(21) $$L_{\upsilon }=\frac{1}{N}{\sum_{n=1}^{N}\left(\upsilon_{\mathrm{state}}-\upsilon_{\mathrm{phy}}\right)}^{2}$$

This term requires that the crosslinking density state learned by the network be as close as possible to the aging kinetics, thereby establishing a structured relationship among “aging kinetics—crosslinking density state representation—viscoelastic parameter response.”

Considering that the thermal aging process has a clear time directionality, this paper also imposes a weak monotonicity constraint on the evolution path of the crosslinking density state. Specifically, after sorting the samples in ascending order of aging time, the loss is defined in Equation (22):

(22) $$L_{\mathrm{mono}}=\frac{1}{N_{t}-1}\sum_{i=1}^{N_{t}-1}{\left[\max(0,-(\upsilon_{\mathrm{state}}^{i+1}-\upsilon_{\mathrm{state}}^{i}))\right]}^{2}$$

This term only penalizes cases where the internal state decreases with increasing time, and is therefore a soft monotonicity constraint. Its purpose is not to require strict monotonicity for all output parameters, but rather to ensure that the crosslinking density state learned by the network generally follows the direction of thermal aging evolution, thereby reducing disorderly oscillations that may occur under small-sample conditions.

In summary, the loss function system of PIMANO is not a mechanical superposition of several penalty terms, but corresponds to its network structure in a one-to-one manner: $L_{\mathrm{data}}$ ensures multi-output prediction accuracy; $L_{e}$ and $L_{\tau }$ constrain the physical admissibility of the constitutive parameters; $L_{\upsilon }$ reinforces the consistency of the crosslinking density state; and $L_{\mathrm{momo}}$ ensures the reasonableness of the aging path direction. What PIMANO optimizes is not merely the minimization of statistical error, but a joint balance among “accuracy, bridging state consistency, and physical admissibility.” For the problem of solid propellant aging, this design aligns more closely with the requirements of material modeling than simply pursuing a reduction in average error, as the latter may come at the cost of physical plausibility.

## 4. Results and Discussion

### 4.1. RBF Data Augmentation

The raw thermal aging data of solid propellants originate from accelerated thermal aging experiments, stress relaxation experiments, and swelling method tests. The sampling points are mainly distributed across limited temperature levels and discrete aging durations, exhibiting typical characteristics such as small sample size, non-uniform sampling, and local sparsity. For such material data, if a data-driven model is trained directly, the model is often affected by insufficient coverage of discrete samples, manifesting as unstable local fitting, amplified interpolation errors across intervals, and increased sensitivity to noise. Therefore, the RBF method is employed to construct a continuous augmented field for crosslinking density and each viscoelastic parameter in the temperature–time two-dimensional space, providing a smoother, more continuous, and structured input foundation for subsequent physical modeling and operator learning. The RBF interpolation used a linear kernel with shape parameter *ε* = 1.0. The smoothing parameter is set to 0.0 to achieve exact interpolation. For neighborhood selection, a global interpolation scheme is used, where all training sample points are invoked for each prediction point.

During the augmentation process, a positive lower bound control, anomaly prediction correction, and physical range clipping for crosslinking density are introduced to avoid obvious non-physical fluctuations in regions with low sample density. Particularly for crosslinking density, the augmented results are constrained near the reasonable fluctuation range of the experimental data, thereby reducing the adverse impact of “interpolation overshoot” on subsequent physical modeling. This treatment is especially important for small-sample material problems, because if systematic pseudo-structures are introduced during the augmentation stage, even the most sophisticated subsequent models may learn incorrect spatial patterns.

**Figure 5:**
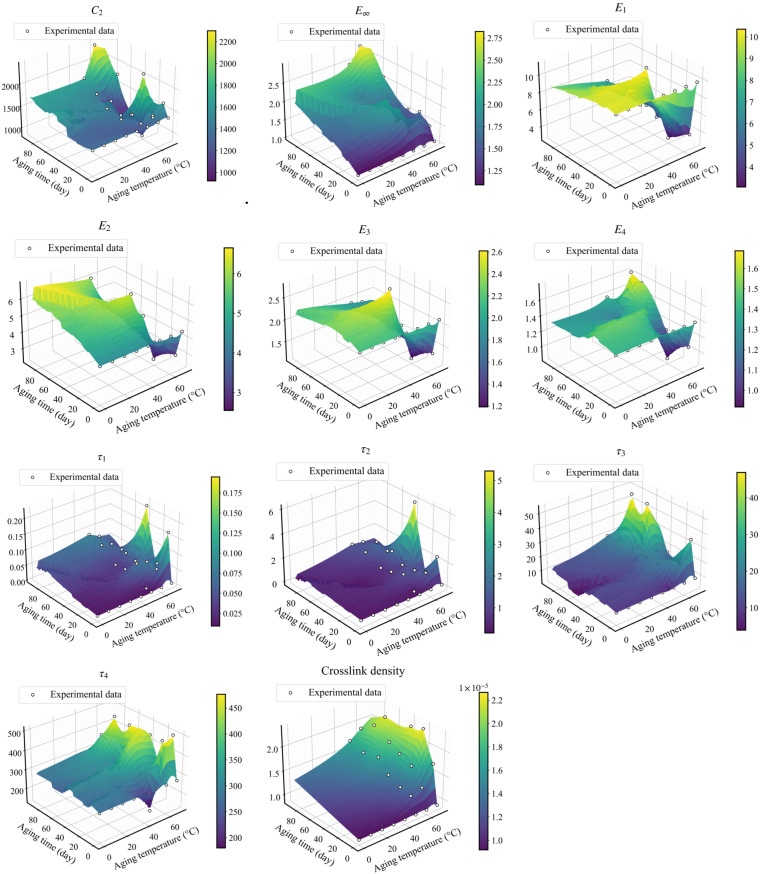
The continuous response distribution of the variables after RBF augmentation in the temperature–time plane.

Figure 5 illustrates the continuous response distribution of the variables after RBF augmentation in the temperature–time plane. It can be seen that the experimental points and the augmented field remain consistent in the main trend, indicating that the interpolation results do not deviate from the support of the original observations. Meanwhile, the augmented field forms a smooth transition between adjacent experimental points, providing the necessary conditions for constructing local neighborhood state vectors in the neural operator model.

### 4.2. Reduced-Order Model Evaluation

After constructing the continuous state field, further consideration is given to whether there exists an exploitable low-dimensional intrinsic structure within the high-dimensional observation space of the material aging response. Since the sample inputs include not only aging temperature, aging time, and crosslinking density, but the corresponding outputs are also multi-parameter coupled viscoelastic responses, modeling directly in the original variable space can easily lead to the model over-relying on local fluctuations in the limited samples, making it difficult to fully capture the dominant evolution patterns along the aging path. Therefore, three methods—PCA, Isomap, and Autoencoder—are respectively adopted in this paper to construct reduced-order representations, and the resulting latent variables are used as additional inputs to the trunk network to evaluate the role of low-dimensional structures in material aging modeling.

**Figure 6:**
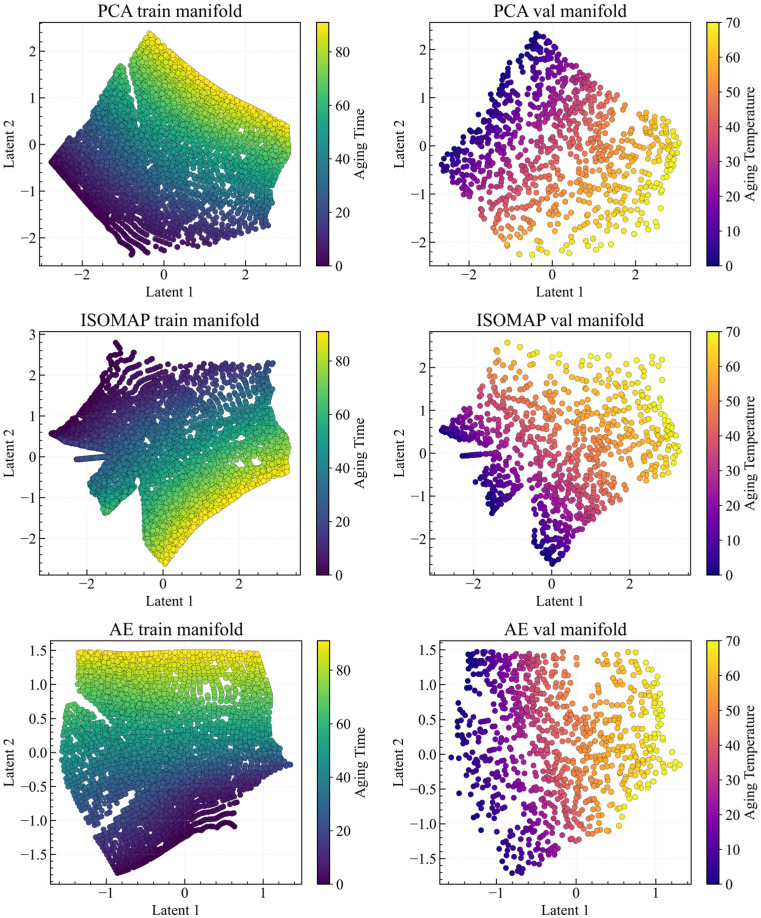
The latent space distribution of aging samples obtained by different reduced-order methods.

Figure 6 shows the two-dimensional latent space distributions obtained by the three reduced-order methods on the training and validation sets. In terms of overall trends, all three methods can organize the aging samples into low-dimensional trajectories that exhibit temporal progression to some extent, but significant differences exist in their ability to preserve local structures and global geometry. PCA tends to retain the principal axes of global variance, providing certain stability in sample ordering, but it insufficiently captures locally curved trajectories. Isomap better reflects the curved separation relationships among different temperature paths, indicating stronger sensitivity to the nonlinear geometry of the material state manifold. Autoencoder forms a more flexible local distribution in the latent space, but under small-sample conditions it also presents a higher risk of overfitting to local structures.

To quantitatively evaluate the effectiveness of the three reduced-order methods, a comprehensive assessment is conducted using metrics such as trustworthiness, pairwise distance correlation, and reconstruction error. Trustworthiness measures the degree of local neighborhood preservation after dimensionality reduction. Distance correlation reflects the ability of the low-dimensional embedding to inherit the geometric structure of the original space. Reconstruction error is primarily applicable to models with an explicit inverse mapping, such as PCA and Autoencoder. Table 4 summarizes the quantitative evaluation results of the three reduced-order methods.

As shown in Table 4, if PCA outperforms the other methods in both trustworthiness and pairwise distance correlation, it indicates that PCA is more effective in preserving both the local relationships and the global geometry of the aging path. For the problem addressed in this paper, such a low-dimensional structure is not an end in itself but rather serves to provide a more organized trunk representation for the neural operator. The value of reduced-order methods lies not in forming a prediction model on their own, but in helping the main model, PIMANO, better identify the state positions along the aging trajectory. Ultimately, the advantage of the main model is determined by the synergistic integration of the low-dimensional manifold structure, bridging state constraints, local-state field sampling, and the neural operator mechanism.

### 4.3. Model Comparison and Analysis

To validate the effectiveness of PIMANO, this paper systematically compares it with Traditional, PINN, DeepONet, and manifold-enhanced DeepONet. Among these, Traditional represents a low-complexity statistical regression baseline, PINN represents an explicit physics-regularized neural network baseline, DeepONet represents a standard operator learning baseline, and DeepONet-pca, DeepONet-isomap, and DeepONet-ae are used to examine the gains obtained by introducing only the manifold structure. Unlike these models, the core feature of PIMANO lies in simultaneously integrating the local-state field, manifold latent variables, and a physical bridging state encoding module. Therefore, it is not a simple matter of “adding a few more inputs,” but rather redefines the structure of material aging modeling at the level of model organization.

The RBF-augmented samples were divided into training and validation subsets at an 80%/20% ratio before neural-network training. The split was performed at the sample-coordinate level in the temperature–aging-time domain, and the two subsets were mutually exclusive. Validation samples were not used for gradient updating, loss minimization, or model-parameter optimization. Therefore, no identical RBF-augmented sample was shared between the training and validation sets. The validation results based on the RBF-augmented data should be interpreted as interpolation performance within the reconstructed aging field, rather than fully independent external validation.

**Figure 7:**
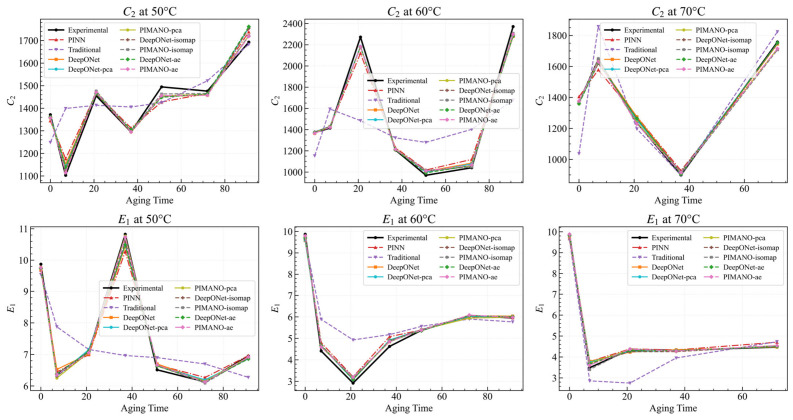

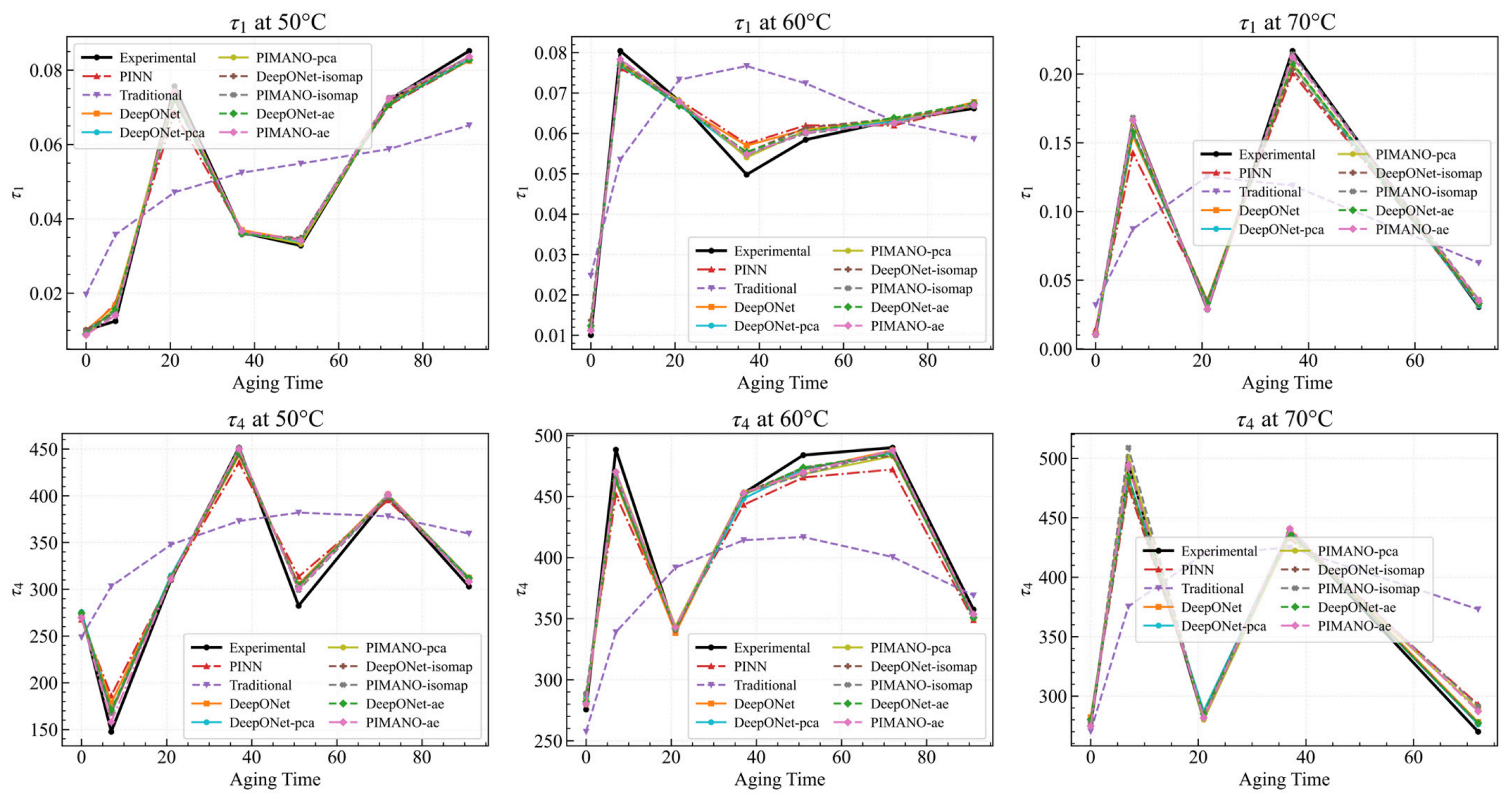
Comparison of fitting results of typical variables under different models with experimental data.

Figure 7 presents the fitting results of several representative variables for different models against experimental data. Using experimental observations at typical aging temperatures as a reference, the figure compares the tracking capabilities of Traditional, PINN, DeepONet, and PIMANO on parameter evolution curves.

Table 5 compares the average prediction performance of different models under two data settings: RBF-augmented data and original experimental data. The results clearly demonstrate the importance of RBF augmentation for small-sample aging prediction. When only the original experimental data were used, all neural-network-based models exhibited relatively large prediction errors, with RMSE values ranging from 45.26 to 67.21 and MAE values ranging from 32.38 to 47.92. Moreover, several models showed low or even negative R-square values, indicating unstable fitting and poor generalization under sparse experimental sampling.

After introducing RBF augmentation, the prediction accuracy of all neural-network-based models improved substantially. For example, PIMANO-ae achieved RMSE = 0.7847, MAE = 0.3366, R^2^ = 0.9995, and MRE = 0.0027 under the RBF-augmented setting. Compared with the original experimental data setting, the RMSE, MAE, and MRE of PIMANO-ae were reduced by 98.31%, 99.08%, and 99.20%, respectively. Similar improvements were also observed for PIMANO-pca and PIMANO-isomap, confirming that RBF augmentation effectively alleviates the sparsity of the original aging dataset and provides a smoother representation of the temperature–aging-time state space.

Among all RBF-augmented neural-network-based models, the PIMANO variants achieved the best overall performance. Specifically, PIMANO-pca obtained the lowest RMSE of 0.7805, while PIMANO-ae achieved the lowest MAE and MRE values of 0.3366 and 0.0027, respectively. The PIMANO variants also maintained the highest R-square values, reaching 0.9995. These results suggest that manifold-based representations can effectively capture the nonlinear evolution of aging-induced material-property changes. Compared with DeepONet, PINN, and the traditional model, PIMANO benefited not only from data enrichment by RBF interpolation but also from the incorporation of manifold learning and physics-informed constraints.

These results indicate that RBF augmentation is a critical component of the proposed framework. It does not merely increase the number of training samples, but reconstructs a continuous and physically smooth aging-state field from sparse experimental observations. This enriched state representation improves the stability of neural-operator training and enables more accurate interpolation within the investigated aging domain.

**Figure 8:**
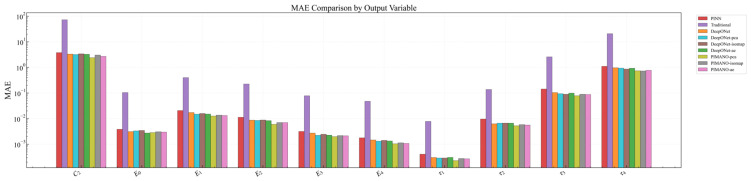
Mean Relative Error (MRE) of each output variable across different models.

As can be seen from the average relative errors for each output variable across different models in Figure 8, PIMANO-ae achieves the lowest MAE for the relaxation time parameters τ_1_–τ_4_, making it the best-performing model for these outputs. Considering all output variables collectively, the PIMANO family of models performs the best overall, surpassing traditional models, PINN, and other DeepONet.

### 4.4. Validation of Physical Loss Term Effectiveness

To further evaluate the contribution of different physical constraint terms to the prediction performance of the PIMANO-ae model, this paper designs ablation experiments on physical constraints. The experiment uses a model that includes only the data-driven loss as the baseline, and separately examines the impact of the full physical constraints, the relaxation time non-negativity constraint, the modulus non-negativity constraint, the crosslink-density physical constraint, and the crosslink-density smoothness constraint on model performance. The evaluation metrics include average RMSE, MAE, R^2^, and MRE, and the results are shown in Table 6.

It can be observed that the full physical constraints model achieves the best overall performance, with Avg_RMSE and Avg_MAE of 0.7828 and 0.3364, respectively; both lower than those of the data_only model that incorporates only the data-driven loss. Meanwhile, the full constraints model attains an Avg_R^2^ of 0.99956 and an Avg_MRE of 0.00278, indicating high prediction accuracy and stability.

Compared with the single-physical-constraint models, the full_constraints model is generally superior in terms of error metrics. For example, although tau_nonnegative_only and physics_c_only exhibit performance close to that of the full constraints model, their Avg_RMSE values are still slightly higher, at 0.7844 and 0.7837, respectively. This suggests that different physical constraint terms have a certain complementary effect, and the full constraints can more comprehensively introduce physical prior information from the material aging process.

### 4.5. Weight Selection and Sensitivity Analysis for Physical Loss Functions

To avoid relying on manual experience for setting the weights of physical loss functions, this paper adopts a data-driven optimization approach for the key physical constraint weights in the PIMANO-ae model. Based on the model architecture and the aforementioned settings, only four physical loss weights are subject to search, optimization, and sensitivity validation: the relaxation time non-negativity constraint weight *λ_τ_*, the modulus non-negativity constraint weight *λ_e_*, the crosslink-density physical consistency constraint weight *λ_υ_*, and the crosslink-density smoothness constraint weight *λ*_momo_.

We generate candidate weight combinations on a logarithmic scale and perform rapid screening using a small training budget. The weight combination that achieves the best comprehensive performance in the rapid screening is selected, and the model is retrained with the full training budget. The final weights are determined based on a comprehensive selection metric on the validation set. This comprehensive selection metric is defined in Equation (23):

(23) $$\begin{array}{c} \mathrm{Score}=0.6(1-R^{2})+0.25\log_{10}(1+\mathrm{MRE})+\\ \;\;\;\;\;\;\;\;\;\;\;\;\;0.1\log_{10}(1+\mathrm{RMSE})+0.05\log_{10}(1+\mathrm{MAE}) \end{array}$$

**Figure 9:**
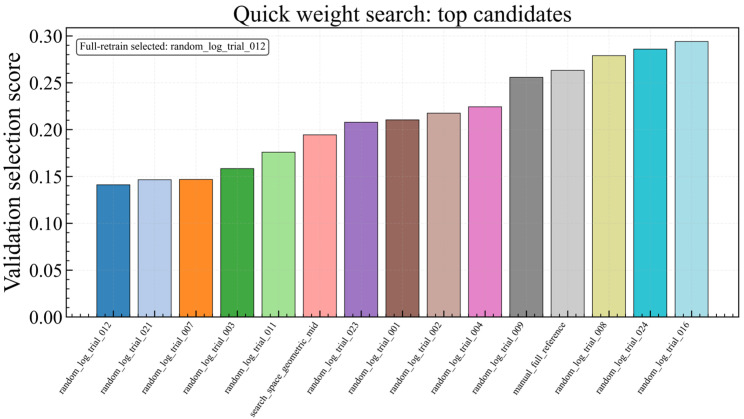
Ranking of candidate model performance on the validation set during rapid weight search.

This metric primarily uses the mean coefficient of determination R^2^, while also taking into account the MRE, RMSE, and MAE. The results are shown in Figure 9. A smaller Score indicates better comprehensive prediction performance of the model on the validation set. The final selected physical loss function weights are shown as Equation (24):

(24) $$\lambda_{\tau }=1.603\times 10^{-5},\lambda_{e}=1.434\times 10^{-4},\lambda_{\upsilon }=2.27\times 10^{-5},\lambda_{\mathrm{mono}}=2.27\times 10^{-6}$$

From the complete retraining results, it can be seen that the optimal weight combination does not simply increase all physical constraint terms, but rather strikes a balance between data fitting error and physical consistency constraints.

To further verify the stability of the final weight selection, we conduct a sensitivity analysis using the one-factor perturbation method. Specifically, while keeping the other three weights unchanged, each weight is adjusted to 0.25, 0.5, 1, 2, and 4 times its final selected value. The model is then retrained, and the changes in the validation set Selection Score, average RMSE, and average R^2^ are recorded. It should be noted that the sensitivity analysis uses an independent retraining budget, so its baseline metrics differ slightly from those in the full-budget retraining stage; we focus mainly on the relative trends of the performance metrics under different perturbation multiples. The results are shown in Table 7.

The sensitivity analysis shows that the model is most sensitive to *λ*_mono_ and *λ_τ_*, indicating that the crosslink-density smoothness constraint and the relaxation time non-negativity constraint have the greatest impact on model stability. When these two weights are too small, the model’s Score and RMSE increase significantly, while R^2^ decreases markedly, suggesting that insufficient physical constraints weaken the model’s ability to represent the aging law and the physical feasibility of Prony parameters. Conversely, when the weights are too large, the balance between data fitting and physical constraints may also be affected.

In comparison, *λ_υ_* mainly affects model performance when the weight is too small, indicating that the crosslink-density physical consistency constraint helps improve model generalization ability but is relatively robust over a wide range. *λ_e_* has almost no effect on the results, suggesting that the modulus parameters predicted by the current model already essentially satisfy the non-negativity requirement, and this constraint primarily serves an auxiliary stabilizing role.

### 4.6. Temperature Leave-One-Out Cross-Validation

To evaluate the generalization ability of the proposed PIMANO-ae model under different thermal aging temperatures, this paper adopts a temperature leave-one-out cross-validation strategy. In each fold, one aging temperature is completely excluded from the training set and used as an independent validation temperature; samples at the remaining temperatures are used for model training. Specifically, 50 °C, 60 °C, and 70 °C are successively held out as the left-out temperature for cross-validation. For each fold, the primary validation set consists of all raw experimental samples and interpolated samples at the held-out temperature; in addition, the subset of raw experimental samples at that temperature is separately used for diagnostic evaluation and visualization.

Model performance is evaluated using RMSE, MAE, R^2^, and MRE. These metrics are first computed for each output variable, including *C*_2_, *E*_∞_~*E*_4_, and *τ*_1_~*τ*_4_, and then averaged across all output variables to obtain the comprehensive validation performance at each held-out temperature. The average validation results are shown in Table 8.

The temperature leave-one-out cross-validation results indicate that the PIMANO-ae model achieves good prediction accuracy and stable generalization ability under unseen aging temperature conditions. For the three held-out temperatures of 50 °C, 60 °C, and 70 °C, the model’s average R^2^ values are 0.9469, 0.9647, and 0.9554, respectively, all exceeding 0.94; the corresponding average MRE values are 6.21%, 4.98%, and 5.26%, demonstrating that the model can accurately predict the evolution of Prony series parameters with aging time at different temperatures.

**Figure 10:**
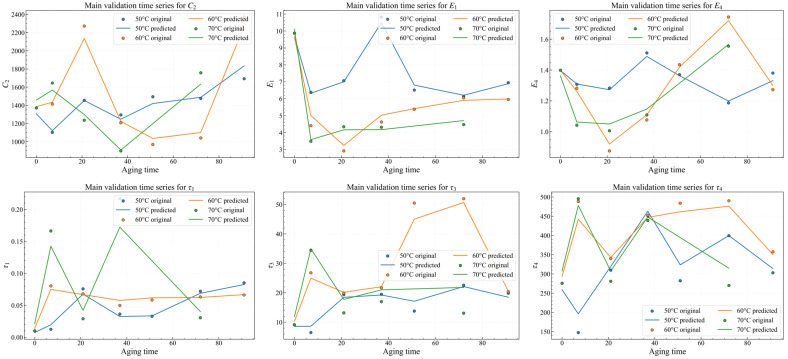
Results of selected parameters under temperature leave-one-out cross-validation.

Figure 10 shows the results for selected parameters under temperature leave-one-out cross-validation. The results indicate that PIMANO-ae not only fits the aging behavior at known temperatures but also possesses strong extrapolation capability under unseen temperature conditions, providing a reliable method for modeling the viscoelastic Prony series parameters during the aging process of solid propellants.

### 4.7. Dimensionality Sensitivity Analysis

To evaluate the impact of the latent space dimensionality on the predictive performance and generalization ability of the PIMANO model, this paper examines the model performance of three dimensionality reduction methods—PCA, Isomap, and AE—under latent dimensionalities of 1, 2, and 3. The results are shown in Table 9.

The results show that different dimensionality reduction methods exhibit significant differences in sensitivity to latent dimensionality, but overall, the model maintains high prediction accuracy under all dimensionality settings, with average R^2^ exceeding 0.999, indicating that the PIMANO framework possesses strong fitting capability and stability. For AE, both two-dimensional and three-dimensional configurations perform excellently, but the two-dimensional AE has advantages in terms of RMSE, MAE, and generalization gap. Therefore, if minimizing overall error and achieving model robustness are the primary goals, PIMANO-ae-d2 can be recommended as the configuration of choice; if relative error, coefficient of determination, and validation loss are of greater concern, then PIMANO-ae-d3 is also a competitive alternative. Overall, compared with PCA and Isomap, the AE dimensionality reduction method can more fully extract nonlinear low-dimensional features and is therefore a more suitable latent representation for the PIMANO model in this study.

Taken together, PIMANO consistently outperforms the corresponding DeepONet variants under comparable manifold representations, indicating that its improvement does not arise from dimensionality reduction alone, but from the synergistic integration of physical bridge-state encoding, local-state-field representation, manifold latent coordinates, and the neural-operator backbone. Compared with the Traditional baseline, which mainly relies on empirical regression, and PINN, which mainly introduces physics-based regularization, PIMANO establishes a more coherent relationship among the aging-path position, local neighborhood state structure, and crosslink-density bridge constraint. This enables the model to better capture the complex coupling between crosslinked-network evolution and multi-parameter viscoelastic responses during thermal aging of HTPB solid propellants. The ablation and temperature leave-one-out validation results further suggest that the physical constraints are not merely auxiliary regularization terms, but also contribute to maintaining the physical feasibility of the predicted Prony-series parameters and the stability of cross-temperature generalization. Therefore, the multi-parameter response of thermally aged solid propellants should not be simplified as a planar mapping directly determined by temperature and time; rather, it should be regarded as a state-dependent operator mapping jointly governed by internal bridge-state evolution, local-state-field morphology, and global aging-manifold position. The overall superiority of PIMANO in accuracy, stability, and physical interpretability demonstrates its potential as a reliable methodological basis for aging-state prediction, life assessment, and service reliability analysis of solid propellants.

## 5. Conclusions

This paper proposes a physics-informed manifold neural operator model, termed PIMANO, for multi-parameter prediction of thermal aging in HTPB solid propellants. The method is built upon experimental data including aging temperature, aging time, crosslinking density, and viscoelastic Prony-series parameters. A continuous aging-state field is constructed using RBF interpolation, and crosslinking density is elevated from an ordinary input variable to a physically interpretable bridge-state variable connecting aging kinetics with viscoelastic response prediction. Among the three candidate aging kinetic models, the modified Arrhenius–Avrami model gives the best quantitative fitting performance for crosslinking-density evolution, with R^2^ = 0.988 and MRE = 0.0199, outperforming the double-exponential competition model and the Arrhenius–Avrami model.

The main contribution of this work lies in the integration of bridge-state modeling, manifold representation, local-state-field sampling, and neural-operator learning into a unified prediction framework. Quantitative comparison with Traditional, PINN, DeepONet, and manifold-enhanced DeepONet models demonstrates that PIMANO provides superior prediction accuracy under the RBF-augmented validation setting. Specifically, PIMANO-ae achieves RMSE = 0.7847, MAE = 0.3366, R^2^ = 0.9995, and MRE = 0.0027, while PIMANO-pca obtains the lowest RMSE of 0.78059. Compared with the traditional model under the same RBF-augmented setting, PIMANO-ae reduces RMSE from 15.47 to 0.7847, MAE from 9.5413 to 0.3366, and MRE from 0.08581 to 0.0027, corresponding to reductions of 94.93%, 96.47%, and 96.85%, respectively. Compared with the original experimental data setting, the RBF-augmented PIMANO-ae further reduces RMSE, MAE, and MRE by 98.31%, 99.08%, and 99.20%, respectively, indicating that the reconstructed continuous aging-state field effectively alleviates the sparsity of the experimental dataset.

The ablation study further confirms the effectiveness of the physical constraint terms. The full-constraint PIMANO-ae model achieves average RMSE = 0.7828, average MAE = 0.3364, average R^2^ = 0.99956, and average MRE = 0.00278, showing slightly better overall performance than the data-only model and single-constraint variants. The sensitivity analysis indicates that the model is most sensitive to the crosslink-density smoothness constraint and the relaxation-time non-negativity constraint, whose perturbations lead to maximum RMSE changes of 347.88% and 269.49%, respectively. These results suggest that the physical constraints do not merely act as auxiliary regularization terms, but contribute to maintaining the stability and physical feasibility of the predicted Prony-series parameters.

Furthermore, the temperature leave-one-out cross-validation verifies the generalization ability of PIMANO-ae under unseen aging temperature conditions. When 50 °C, 60 °C, and 70 °C are respectively held out from training, the model obtains average R^2^ values of 0.9469, 0.9647, and 0.9554, and corresponding average MRE values of 6.21%, 4.98%, and 5.26%. These results demonstrate that PIMANO-ae can not only interpolate within the reconstructed aging field, but also maintain stable predictive performance across unseen thermal aging temperatures. Overall, the proposed PIMANO framework provides a quantitative, physically informed, and interpretable modeling strategy for solid propellant aging prediction, and may offer a useful reference for state prediction and life assessment of other aging polymer-based energetic materials.

## Figures and Tables

**Table 1:** Sampling time under different aging temperatures.

Aging Temperature (°C)	Sampling Time (Days)
50 °C	[0, 7, 21, 37, 51, 72, 91]
60 °C	[0, 7, 21, 37, 51, 72, 91]
70 °C	[0, 7, 21, 37, 72]

**Table 2:** Material parameters of HTPB propellant.

Aging Temperature (°C)	Sampling Time (Days)	*C*_1_	*C*_2_ (°C)	*E*_∞_ (MPa)	*E*_1_ (MPa)	*E*_2_ (MPa)	*E*_3_ (MPa)	*E*_4_ (MPa)	*τ*_1_ (s)	*τ*_2_ (s)	*τ*_3_ (s)	*τ*_4_ (s)	Crosslinking Density (mol·cm^−3^)
50 °C	0	50	1371.4	1.1	9.8	4.4	2.1	1.4	0.010	0.30	9.1	275.6	9.12 × 10^−6^
	7	50	1103.0	1.6	6.3	4.1	1.7	1.3	0.012	0.28	6.5	147.6	1.24 × 10^−5^
	21	50	1455.3	2.1	7.1	5.4	2.0	1.3	0.075	1.21	19.3	309.8	1.30 × 10^−5^
	37	50	1294.9	2.1	10.8	6.3	2.7	1.5	0.036	0.84	19.5	451.5	1.42 × 10^−5^
	51	50	1494.5	1.6	6.5	4.7	1.9	1.3	0.032	0.67	13.7	282.4	1.67 × 10^−5^
	72	50	1475.8	1.7	6.1	4.6	1.8	1.1	0.072	1.27	22.5	399.5	1.60 × 10^−5^
	91	50	1692.9	2.4	6.9	6.0	1.9	1.4	0.085	1.30	19.8	302.9	1.70 × 10^−5^
60 °C	7	50	1414.1	1.7	4.4	3.3	1.7	1.3	0.080	1.46	26.7	488.4	1.32 × 10^−5^
	21	50	2272.4	1.2	2.9	2.5	1.1	0.8	0.068	1.16	19.9	339.9	1.65 × 10^−5^
	37	50	1208.6	1.3	4.6	3.6	1.5	1.1	0.049	1.03	21.7	452.6	1.77 × 10^−5^
	51	50	969.5	1.6	5.3	3.5	1.5	1.4	0.058	1.71	50.5	483.8	1.90 × 10^−5^
	72	50	1040.5	2.2	6.1	4.2	1.9	1.7	0.063	1.81	51.9	490.0	2.09 × 10^−5^
	91	50	2371.5	2.8	5.9	4.0	1.8	1.2	0.066	1.16	20.4	357.5	1.88 × 10^−5^
70 °C	7	50	1645.5	1.4	3.4	2.8	1.3	1.0	0.166	2.39	34.4	495.6	1.72 × 10^−5^
	21	50	1236.5	1.6	4.3	3.2	1.3	1.0	0.029	0.61	13.1	280.8	2.32 × 10^−5^
	37	50	899.0	1.5	4.3	2.4	1.3	1.1	0.21	5.90	16.9	438.9	2.25 × 10^−5^
	72	50	1757.9	2.1	4.4	4.4	1.8	1.5	0.030	0.63	13.1	270.0	2.18 × 10^−5^

**Table 3:** Fitting metrics of the three traditional models.

Traditional Model	R^2^	MRE
Modified Arrhenius–Avrami	0.988	0.0199
Double-exponential Competition	0.985	0.0257
Arrhenius_Avrami	0.951	0.0364

**Table 4:** Quantitative evaluation results of different reduced-order methods.

Method	Trustworthiness	Pairwise Distance Correlation	Reconstruction RMSE
PCA	0.9998	0.9982	2.995
Isomap	0.9996	0.9932	\
Autoencoder	0.9999	0.9451	0.2394

**Table 5:** Average prediction performance of different models under RBF-augmented and original experimental data settings.

Model	Data Setting	RMSE	MAE	R^2^	MRE
PIMANO-ae	RBF-augmented	0.7847	0.3366	0.9995	0.0027
	Original experimental	46.51	36.47	0.3913	0.3362
PIMANO-pca	RBF-augmented	0.78059	0.3544	0.9995	0.0028
	Original experimental	47.52	34.95	0.4093	0.3069
PIMANO-isomap	RBF-augmented	0.8137	0.3695	0.9995	0.0029
	Original experimental	50.07	36.32	0.2973	0.2986
DeepONet-ae	RBF-augmented	0.8448	0.4032	0.9994	0.0032
	Original experimental	45.26	32.38	−0.1878	0.3271
DeepONet-pca	RBF-augmented	0.8542	0.4059	0.9994	0.0032
	Original experimental	54.57	43.56	−0.0507	0.3214
DeepONet-isomap	RBF-augmented	0.8805	0.4213	0.9993	0.0034
	Original experimental	55.99	37.81	0.1105	0.2876
DeepONet	RBF-augmented	0.9751	0.4333	0.9992	0.0037
	Original experimental	56.31	40.61	0.0510	0.2593
PINN	RBF-augmented	1.3263	0.5411	0.9986	0.0046
	Original experimental	67.21	47.92	0.2580	0.3462
Traditional	RBF-augmented	15.47	9.5413	0.7717	0.08581
	Original experimental	64.48	49.15	−0.0510	0.3969

**Table 6:** Ablation results of different physical constraints on the prediction performance of the PIMANO-ae model.

Model	Avg_RMSE	Avg_MAE	Avg_R^2^	Avg_MRE
full_constraints	0.7828	0.3364	0.99956	0.00278
data_only	0.7911	0.3387	0.99956	0.00278
*L_e_*_only	0.7939	0.3426	0.99955	0.00282
*L_τ_*_only	0.7844	0.3370	0.99956	0.00278
*L_υ_*_only	0.7837	0.3373	0.99956	0.00278
*L*_mono__only	0.7907	0.3415	0.99955	0.00281

**Table 7:** Sensitivity analysis results of regularization hyperparameters.

Weight Parameter	Baseline Weight	Maximum Relative Change in Score	Maximum Relative Change in RMSE	Maximum Relative Change in R^2^
*λ*_mono_	2.172 × 10^−6^	153.18%	347.88%	3.64%
*λ* *_τ_*	1.603 × 10^−5^	146.26%	269.49%	3.66%
*λ* *_υ_*	2.270 × 10^−5^	112.98%	219.84%	2.37%
*λ* *_e_*	1.434 × 10^−4^	101.46%	153.18%	1.03%

**Table 8:** Average validation performance of the PIMANO-ae model under temperature leave-one-out cross-validation.

Leave-One Temperature (°C)	Avg_RMSE	Avg_MAE	Avg_R^2^	Avg_MRE
50	9.6022	7.3030	0.9469	6.21%
60	9.8829	7.8517	0.9647	4.98%
70	6.3456	4.5551	0.9554	5.26%

**Table 9:** Dimensionality sensitivity analysis results of the PIMANO model under different latent dimensionalities.

Model	Latent Dimension	Avg_RMSE	Avg_MAE	Avg_R^2^	Avg_MRE
PIMANO-ae	1	0.9633	0.4578	0.9991	0.00379
	2	0.9271	0.4326	0.9993	0.00349
	3	0.9504	0.4382	0.9993	0.00341
PIMANO-isomap	1	1.0110	0.4709	0.9992	0.00364
	2	0.9963	0.4933	0.9992	0.00366
	3	1.0809	0.5231	0.9991	0.00413
PIMANO-pca	1	0.9776	0.4499	0.9992	0.00361
	2	0.9650	0.4512	0.9993	0.00355
	3	1.0152	0.4854	0.9992	0.00370

## Data Availability

The original contributions presented in this study are included in the article. Further inquiries can be directed to the corresponding author.

## Abbreviations

The following abbreviations are used in this manuscript:

PIMANO	Physics-Informed Manifold Neural Operator
HTPB	Hydroxyl-Terminated Polybutadiene
AP	Ammonium Perchlorate
RBF	Radial Basis Function
PINN	Physics-Informed Neural Network
DeepONet	Deep Operator Network
WLF	Williams–Landel–Ferry
PCA	Principal Component Analysis
Isomap	Isometric Mapping
AE	Autoencoder
MDS	Multidimensional Scaling
RMSE	Root Mean Square Error
MAE	Mean Absolute Error
MRE	Mean Relative Error
R^2^	Coefficient of Determination
GJB	Chinese Military Standard
QJ	Aerospace Industry Standard of China
NMR	Nuclear Magnetic Resonance
MSE	Mean Squared Error
